# Is a Bimodal Force-Time Curve Related to Countermovement Jump Performance?

**DOI:** 10.3390/sports6020036

**Published:** 2018-04-18

**Authors:** Rodney A. Kennedy, David Drake

**Affiliations:** 1 School of Sport, Ulster University, Jordanstown, Shore Road, Newtownabbey, County Antrim BT37 0QB, UK; 2 Ulster Rugby, Kingspan Stadium, Belfast BT6 0FT, UK; daviddrake87@gmail.com

**Keywords:** movement, attention, neuromuscular function, shape

## Abstract

A countermovement jump (CMJ) represents one of the most frequently used performance tests for monitoring neuromuscular function in athletes. An often-overlooked feature that may provide some useful diagnostic information is the actual shape of the force-time curve. The aim of this study was therefore to consider how the shape of the force-time curve influences jump performance. Thirty-three male rugby union players performed two CMJs on a force plate, with discrete variables and continuous curve analysis used. The subjects were dichotomized based on shape of the force-time curve during the propulsion phase and by jump height. The differences between the unimodal and bimodal groups were unclear for jump height (ES = 0.28, ±0.58) and reactive strength index-modified (ES = −0.30, ±0.59). A substantial difference between high (40.2 ± 2.9 cm) and low (31.2 ± 3.2 cm) jumpers only existed in the late propulsion phase by 79.0% to 97.0% of the normalized force-time curve. A bimodal force-time curve is not representative of an optimal pattern of performance and simply reflects an inefficient stretch-shortening cycle. The inter-individual variability that exists in braking COM displacement renders temporal phase analysis impractical in cross-sectional type studies.

## 1. Introduction

Vertical jumping is a fundamental movement skill that develops during early childhood and is a key component of many sports. Since vertical jumping represents a whole-body movement, it can be purposively constrained in a variety of ways, namely, with and without a countermovement, arm swing or external loading [1]. Nonetheless, an unloaded countermovement jump (CMJ) with no arm swing represents one of the most frequently used performance tests for monitoring neuromuscular function in athletes [2,3]. The subsequent analysis of such testing is typically centred on the main performance variable, jump height, which can be reliably measured using a variety of affordable equipment. However, this somewhat simplistic approach may, on occasion, lack the required capacity to explain the changes observed [4,5] and therefore, rationalises, to some degree, the preponderance of force plate analysis within performance sport.

A force platform can record the ground reaction force-time data at a high resolution during a CMJ, with the velocity and displacement of the centre of mass subsequently calculated using the impulse-momentum theorem. Although the standard approach of selecting a series of discrete variables to explain the jump performance has proven useful [2,3], large amounts of data are invariably disregarded, and this could potentially impair our overall understanding of the area. At the outset, an often-overlooked feature that may provide some useful diagnostic information to the practitioner is the actual shape of the force-time curve [6,7]. In contrast to slight inter-individual differences that may exist in the shape of the unweighting phase [8], two radically different shapes have been consistently observed during the propulsion phase of the jump, a unimodal and a bimodal shape [9,10,11,12,13,14].

Differing opinions have been offered to explain the shape of the force-time curve during the propulsion phase. Preliminary research suggests that incorporating an arm swing during a CMJ would superimpose a second peak onto the latter portion the force-time curve [10]. Conversely, a bimodal curve has been attributed to the force exerted by the trunk, with an arm swing reducing the two peaks and increasing the local minimum that separates them [15]. However, these suggestions seem implausible, given that Dowling and Vamos [9] reported that 54 of 97 subjects displayed unimodal force-time curves during a CMJ that also incorporated an arm swing. Although certainly not universally agreed upon [9,12], an additional viewpoint that merits consideration is that a bimodal curve, consisting of two peaks during the propulsion phase is indicative of a proficient jumper and therefore, an optimal pattern of performance when jumping for height [11,16]. The establishment of a bimodal force-time curve has been observed after 12 weeks of unloaded vertical jump training [14] when analysed using the linear length normalization (LLN) analysis technique [17], and would therefore seem to support this contention.

Normalization of a curve’s amplitude is routinely done by scaling the variable of choice with an appropriate physical constant, such as body mass or leg length [18]. In addition, some type of temporal phase analysis is required to facilitate comparisons between and within individuals, usually by expressing the data as a percentage of the total duration [17]. The LLN technique compresses or expands the time axis so that all jumps have the same length, typically 500 data points [5,14,19,20,21,22]. As with other types of movements, a CMJ will display a sequence of events characterised by peaks, valleys, zero crossings, and so forth. When jump durations are aligned by LLN, temporal differences between points of interest may still exist due to inter-individual variability and are recognised to slightly confound point-by-point comparisons. However, pooling unimodal and bimodal shaped force-time curves, as is routinely completed [5,7,14,18,19,20,21,22], may lead to misinterpretation of the data. An additional concern is that the null-hypothesis test has been the universal approach to inferential statistics in these studies, and as such, a statistically significant difference in a force-time curve may represent an effect that is practically meaningless, or conversely, a non-significant difference may indeed have a meaningful effect [23]. Therefore, the aims of the current study are two-fold: (1) to determine if a bimodal force-time curve represents an optimal pattern of CMJ performance and (2) to consider the discriminatory effect of pooling and stratifying force-time curves based on whether they exhibit a unimodal or bimodal shape during the propulsion phase of a CMJ. As a working hypothesis, we assume that a bimodal force-time curve represents an optimal pattern of CMJ performance and that pooling force-time curves would confound temporal phase analysis.

## 2. Materials and Methods

### 2.1. Subjects

Thirty-three (*n* = 33) male academy players from a professional rugby union club in the United Kingdom volunteered to take part in the study (age 19.7 ± 1.1 years, height 185.6 ± 7.7 cm, mass 98.9 ± 12.6 kg, and jump height 35.3 ± 5.5 cm). To be eligible for inclusion in the study, players had to have been part of the academy squad at the start of the playing season and therefore, familiar with the CMJ testing that is routinely conducted. Exclusionary criteria included players with a known musculoskeletal injury or pain during the time of testing. The weekly training volume of the players was 7–11 h (4–6 h of rugby training, 1–2 h of speed and agility training, plus 2–3 h of gym-based preparation), and one competitive game. Prior to commencement of the study, the players attended a presentation to outline the purpose, benefits, risks and procedures involved in the study. Players provided written informed consent and were free to withdraw from the study at any stage without penalty. The study was approved by the Ulster University Human Research Ethics Committee.

### 2.2. Design

This was a cross-sectional study of rugby union players, which involved two sub-examinations of CMJ performance: (1) a comparison of players who displayed either a unimodal or bimodal force-time curve shape during the propulsion phase, and (2) a comparison of high and low level jumpers, using either a pooled or stratified approach based on the shape of the curve. Discrete variables and continuous curve analysis were used in both sub-examinations to identify any possible differences. To control for the possible influence of circadian rhythms and the phase of training, all testing was performed during a morning period of the competitive season. Subjects were asked to refrain from strenuous exercise for 48 h beforehand. Prior to testing, subjects completed a 10-min standardised warm-up consisting of jogging, dynamic stretching, and several practice jumps of progressively increasing intensity until they felt capable of producing a maximal effort.

### 2.3. Data Collection

CMJ trials were performed on a force plate (Type 9286BA, Kistler AG, Winterthur, Switzerland) that was connected to an A/D convertor (Type 5691A1, Kistler AG, Winterthur, Switzerland). Temporal and vertical ground reaction force (Fz) data were collected at a sampling frequency of 1000 Hz for 5 s using Bioware^®^ software (Version 5.1, Type 2812A, Winterthur, Switzerland). The force plate was zeroed immediately before each trial, and sampling began when the subject was standing still. After approximately 2 s, subjects were instructed to jump as high as possible using a self-determined countermovement depth. Each subject completed 2 trials with 1 min of rest in between, with the average used for further analysis [7].

### 2.4. Data Analysis

The raw Fz data for each jump were exported as text files and analysed using a customized Microsoft Excel^®^ spreadsheet (version 2016, Microsoft Corp., Redmond, WA, USA). Each subject’s body weight was calculated as the average Fz during the first second of the sampling period. The first time the Fz deviated above or below body weight by more than 1.75 times, the peak residual force found during the 1 s body weight averaging period was identified. A backward search was then completed until Fz passed through body weight; this time point was defined as the start of the countermovement. The point of take-off was determined by finding the 0.4 s moving average with the smallest standard deviation in Fz and then taking the peak residual force during this phase as the threshold [24]. The vertical velocity of the centre of mass (COM) was determined using the impulse method. Net impulse was obtained by integrating the net Fz using the trapezoid method from the start of the countermovement and then dividing it by body mass to obtain vertical velocity. Vertical displacement was subsequently determined through integration with velocity. The braking phase was defined as the moment of minimum velocity until the moment of minimum displacement. The propulsion phase was defined as the moment of minimum displacement until the point of take-off [25]. The vertical velocity of the COM at take-off was used to calculate jump height. To normalize for physical size, force and displacement variables were scaled to body mass and leg length, respectively [18]. To examine the shape of the force-time curve, the propulsion phase was divided into three sub-phases of equal duration [26]. A bimodal force-time curve was then subsequently defined as a decrease in force during the first two sub-phases, followed by an increase in the final sub-phase that was larger than the movement threshold. The efficiency of the stretch-shortening cycle was calculated as the ratio of negative impulse to positive impulse (NPI) [9]. The reactive strength index-modified (RSImod) was calculated as the jump height divided by the contraction time [27].

In addition to the discrete CMJ variables, temporal phase analyses of the jumps were conducted using the LLN technique [17,28]. The force-time curve was selected from each individual participant from the start of the countermovement to the take-off time point. The number of samples in each individual curve was then modified to equal 500 samples by appropriately changing the time intervals between samples and resampling the signal. The normalised curve for each sample was then averaged across all subjects, resulting in high-resolution force-time curves. This procedure allowed for force throughout the jump to be compared across groups at each time point.

### 2.5. Statistical Analysis

Descriptive data are presented as means ± SDs. All other variables were log-transformed to reduce bias due to non-uniformity of error, and analysed using Cohen’s effect size (ES) statistic with ±90% confidence intervals (CI) using a customized Microsoft Excel^®^ spreadsheet [29]. The following magnitude thresholds were used for the standardized differences in means: <0.2 = trivial, <0.6 = small, <1.2 = moderate, <2.0 = large, <4.0 = very large and >4.0 extremely large. The percentage likelihood that the true difference between groups was substantially positive, trivial or substantially negative was calculated, and the qualitative probabilistic terms were assigned using the following scale: <0.5%, most unlikely; 0.5–5%, very unlikely; 5–25%, unlikely; 25–75%, possibly; 75–95%, likely; 95–99.5%, very likely; >99.5%, most likely. When the ±90% CI of the ES spanned small (ES ± 0.2) reference values, the effects were reported as unclear [23,30]. Players were dichotomized into high and low performance groups according to the height achieved in a CMJ. To help ensure that the groups contained subjects with differing levels of performance, a classifying threshold was determined using the formula, mean plus 0.2 multiplied by the between subject standard deviation [31,32]. Multiplying the between subjects standard deviation by 0.2 denotes the smallest worthwhile change (SWC) for this particular sample [33]. Within the context of the current study, providing a classifying threshold greater than the group mean ensured that subjects above this value would exhibit superior jumping proficiency [32].

## 3. Results

The absolute braking COM displacement was 33.3 ± 4.6 cm, with a bimodal force-time curve displayed in 17 of the 33 players (51.5%), for each of their two trials. The differences between the unimodal and bimodal groups were unclear for jump height (Table 1), thus requiring additional research with a larger sample to make the effect clear [23,30]. Nonetheless, the qualitative interpretation of the uncertainty associated with the effect (ES = 0.28, ±0.58) is unlikely to be lower, possibly trivial and possibly greater than the unimodal group [30]. The effect (ES = −0.30, ±0.59) is also unclear for the jump height-derived variable, RSImod and is unlikely to be greater, possibly trivial and possibly lower than the unimodal group. However, the bimodal group adopted a movement strategy that utilised durations that were most likely to be longer (ES = 0.56 to 1.16) durations, were most likely to have a greater (ES = 1.13, ±0.50) braking COM displacement, were very likely to have a greater (ES = 0.95, ±0.55) braking minimum velocity and were possibly to very likely to have lower (ES = −0.41 to −0.79) forces, when compared to the unimodal group. This unique movement strategy adopted by the bimodal group is likely to be more (ES = 0.74, ±0.58) inefficient than the unimodal group, as measured by the NPI variable.

When the groups were stratified based solely on jump performance, the differences between the high and low groups were, as expected, most likely to be greater (ES = 1.22 and 1.66) for jump height and RSImod (Table 2). The high group utilised possibly to likely longer (ES = 0.40 and 0.49) unweighting and braking phases, very likely greater (ES = 0.82, ±0.53) braking COM displacements, likely greater (ES = 0.45, ±0.59) braking minimum velocities and very likely higher (ES = 0.80, ±0.54) mean forces during the propulsion phase, when compared to the low group. This strategy adopted by the high group is likely more (ES = −0.62, ±0.58) efficient than that of the low group, as measured by the NPI variable. Peak propulsion force, unweighting minimum force, braking end force and propulsion phase duration were all unclear.

When the bimodal group was stratified based on jump performance, the differences between the high and low groups were, as expected, very likely and most likely greater (ES = 1.37 and 1.59) for jump height and RSImod (Table 3). The bimodal high group utilised a likely greater (ES = 0.67, ±0.86) braking COM displacement, a likely greater (ES = 0.81, ±0.85) braking minimum velocity and likely to very likely higher (ES = 0.66 to 1.22) forces, when compared to the low group. This strategy adopted by the high group is very likely more (ES = −1.13, ±0.75) efficient than that of the low group, as measured by the NPI variable. All phase durations and the unweighting minimum force were unclear.

When the unimodal group was stratified based on jump performance, the differences between the high and low groups were, as expected, most likely greater (ES = 1.20 and 1.63) for jump height and RSImod (Table 4). The unimodal high group utilised a likely longer (ES = 0.86, ±0.94) unweighting phase, a likely greater (ES = 0.91, ±0.73) braking COM displacement and likely higher (ES = 0.87, ±0.88) mean forces during the concentric phase, when compared to the low group. The strategy adopted by the high group is likely more (ES = −0.78, ±0.98) efficient than that of the low group, as measured by the NPI variable. All other measured variables were deemed unclear.

**Figure 1:**
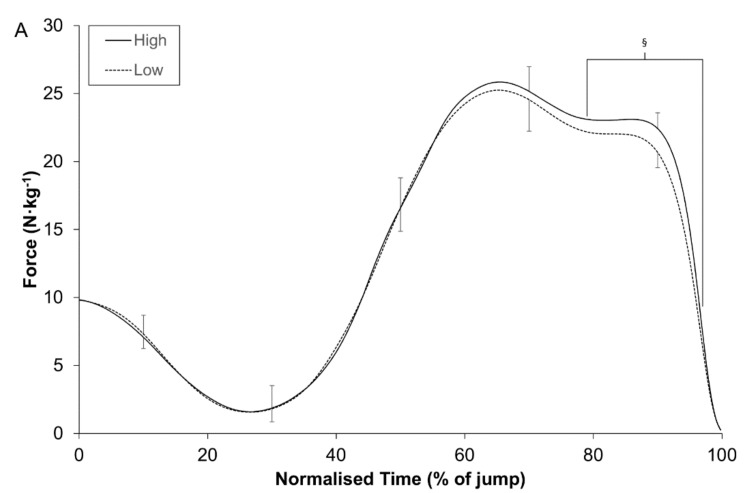

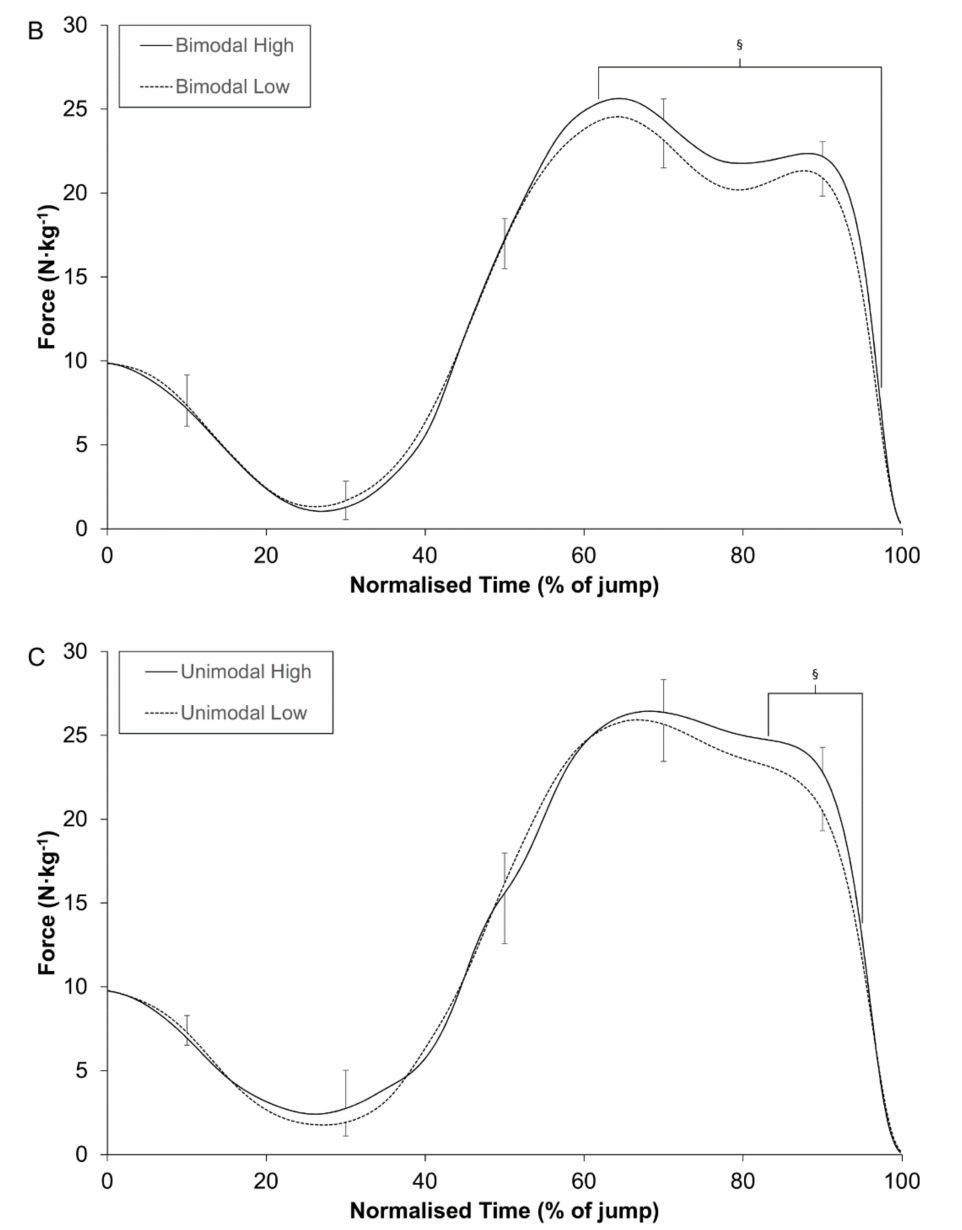
Comparison of the force-time curves between groups: (**A**) high–low; (**B**) bimodal high–low; (**C**) unimodal high-low. Normalised time represents the time from the start of the jump until take-off. § denotes a substantial difference between groups.

The results of the temporal phase analysis revealed that substantial differences between groups existed during the following phases of the CMJ force-time curves: (A) high and low (79.0% to 97.0% of normalised time); (B) bimodal-high and low (61.8% to 97.4% of normalised time); (C) unimodal-high and low (83.2% to 95.0% of normalised time) (Figure 1).

## 4. Discussion

Countermovement jump performance is routinely used to monitor fatigue and supercompensation in athletic populations. Considering that large amounts of data are invariably disregarded when solely focusing on discrete variables, we investigated how the shape of a continuous biomechanical variable, such as force, could provide diagnostic information about jump performance. The main findings of the present study indicate that a bimodal force-time curve does not represent an optimal pattern of CMJ performance and it simply reflects the adoption of a movement strategy that can be characterised as an inefficient stretch-shortening cycle (Table 1). In addition, caution should be exercised when using temporal phase analysis of CMJ force-time curves, as pooling unimodal and bimodal shaped curves in cross-sectional studies may be a confounding factor, due to the force-time profiles of a subgroup that utilise small braking COM displacements during the execution of a CMJ (Figure 1).

When the shape of the force-time curve is considered, the unclear effect for jump height requires additional research with a larger sample to make the effect clear [23,30]. Nonetheless, the qualitative interpretation of the uncertainty associated with the effect (ES = 0.28, ±0.58) does lend support to the findings from previous experimental studies [1,34] that suggest jump height is somewhat insensitive to the braking COM displacement utilised (Table 1). It should, however, be noted that an optimum value for the braking COM displacement does exist and is consistently much greater that the self-selected displacement chosen by subjects, typically by 8–11 cm, on average [1,34]. Such deviations from the self-selected displacement only positively alter the jump height by trivial amounts (<1 cm) and therefore, the weak influence that braking COM displacement has on jump height at these ranges of movement is again clearly illustrated [1,12,13,34,35,36]. This is not to discount the strong influence that braking COM displacement may have on jump height at larger deviations from an optimum value [37].

Vertical jumps are routinely used to test the force production and many other output related capacities of the lower body extensor muscles [18,19]. The results from the present study support the previous literature [1,34,37,38,39] in that braking COM displacement does exert a meaningful influence on kinetic variables, such as propulsion peak force (Table 1). A mechanism that explains this consistent observation relates to the inherent decrease in leverage that parallels the increase in displacement [40]. The higher relative velocities at corresponding joint angles will also contribute to lower forces, due to the well documented force-velocity properties of active muscle [1]. Furthermore, although not always advocated as a CMJ variable [41], power output will also be sensitive to changes in braking COM displacement, primarily influenced by the force part of the equation. Practitioners should therefore be aware that variations in braking COM displacement can erroneously represent a change in performance, when viewed using force as an output variable. Propulsion peak velocity and jump height, unlike many other ancillary variables, are insensitive to modest changes in braking COM displacement and therefore, truly represent actual performance.

Conceptually, it is intuitively appealing to hypothesise that athletes will adopt and should strive for a movement strategy during a CMJ test that reflects their involvement in performance sport, namely, by producing large forces in a time-constrained manner [42]. In other words, athletes have acquired and seek a pattern of force production that may sacrifice a relatively small portion of jump height, by selecting a smaller braking COM displacement, that allows them to successfully complete the task in a shorter duration of time. Unsurprisingly, larger braking COM displacements usually result in longer time frames (Table 1) and are hypothesised to increase the propulsion phase duration to a lesser degree [34], since it is typically shorter in duration. However, due to the work that must be done against gravity, the absolute time differences between groups for the propulsion phase and the combined unweighting and braking phase are identical (35 ms), despite the shorter duration. Nonetheless, as expected, non-athletes do not jump as high, but they do adopt comparable movement strategies to those of athletic populations [34]. Therefore, the movement strategy adopted during a CMJ remains largely unexplained but does not seem to relate to the inherent force production capabilities of an individual (Table 1, Table 2, Table 3 and Table 4) and raises questions about the construct validity of the jump height derived variable, RSImod. Although RSImod has been shown to differentiate between levels of play [42], it would appear to do so predominantly due to the influence of the numerator, jump height. One factor that has been proposed to explain the movement strategy adopted that merits consideration is the range of motion available in the ankle joint, with more flexible individuals adopting a larger braking COM displacement [43]. In addition, the players in this study were given the commonly used neutral instruction, jump as high as possible; this focus of attention will not always maximize CMJ performance and may also partially explain the movement strategies utilised [44].

Temporal phase analysis of a force-time curve is often considered a useful tool to improve our understanding of CMJ performance and is often applied in cross-sectional examinations [14,18]. When the subjects in the current study were stratified by jump height, substantial differences were only identified during the late propulsion phase—79.0% to 97.0% of the normalised force-time curve (Figure 1). Similar findings have been observed in previous studies [14,18] and seem to indicate that normalised force-time curves may lack the required sensitivity to identify differences, despite large differences in jump height. In addition, when subjects have been stratified in other cross-sectional studies by sex [22,45], playing level [20] and dynamic strength index [25] and therefore indirectly by jump height as a consequence, no significant differences in the normalised force-time curves have been observed. It is, however, worth noting that all of these studies adopted a null-hypothesis significance testing approach and this may partially explain some of the findings [23]. Nonetheless, it may be worth reconsidering the usefulness of the LLN analysis technique in cross-sectional studies, as pooling unimodal and bimodal shaped force-time curves is a confounding factor that hampers our understanding of jump performance. The most obvious explanation of the problem relates to a sub-group of players that are classified as having a unimodal force-time curve and a low jumping height. Their movement strategy entails a small braking COM displacement that corresponds with propulsion peak forces that are comparable with more proficient jumpers (Table 4). This scenario ultimately confounds the early portion of propulsion phase in a normalised force-time curve. This sub-group may potentially benefit from feedback, along with an external focus of attention during a CMJ [44], as it likely that their self-selected strategy does not come close to maximising jump height [1]. Furthermore, jump height is determined by the velocity at take-off and is governed precisely by the preceding impulse value, the product of force and time. Although some type of temporal phase analysis is required to facilitate point-by-point comparisons between individuals, usually by expressing movement time as a percentage, this approach is unable to account for small absolute differences in movement time over which force can be applied to alter jump height [20,25,45].

Although this study presents some novel findings, there are a few limitations that the practitioner should keep in mind when interpreting the results. Firstly, increasing the sample size would help to attain a clear effect for the influence of a bimodal force-time curve on jump height, and this study can therefore be viewed as a preliminary investigation into the area. Another limitation to consider is that force-time curves may potentially be sport specific, with the differences observed in the current study being unique to the sport of rugby union [7]. Finally, the sample was dichotomized into only two groups, based on the shape of the force-time curve during the propulsion phase. Further sub-divisions, based on the magnitude of the bimodal force peaks may have provided additional insight [13].

## 5. Conclusions

A bimodal force-time curve does not represent an optimal pattern of performance and it simply reflects the adoption of a movement strategy that can be characterised as an inefficient stretch-shortening cycle. Due to the considerable influence of braking COM displacement, output related variables, such as propulsion peak force should always be interpreted with caution. The factors that can explain the movement strategy adopted during a CMJ are yet to be fully elucidated but may relate to subject characteristics and the instructions provided. Jump height can be considered the key output from a CMJ test; however, the tremendous amount of redundancy that exists in the neuromuscular system permits a multitude of ways in which it can be achieved. Thus, biovariance renders a normalized force-time curve impractical in cross-sectional type studies, due primarily to a sub-group of subjects that adopt a relatively small braking COM displacement when performing a CMJ. The LLN may, however, have merit in training studies, assuming the shape of the curve remains consistent between observations. 

## Figures and Tables

**Table 1:** A comparison of discrete kinetic and kinematic counter movement jump (CMJ) variables between groups by shape.

Jump Variables	Unimodal (*n* = 16)	Bimodal (*n* = 17)	ES (±90% CI)
	Mean ± SD	Mean ± SD	
**Phases**			
Unweighting Phase (ms)	296 ± 22	320 ± 29	0.86, ±0.54 *** ↑
Braking Phase (ms)	146 ± 27	157 ± 15	0.56, ±0.59 ** ↑
Propulsion Phase (ms)	244 ± 28	279 ± 23	1.16, ±0.50 **** ↑
**Forces**			
Propulsion Peak Force (N·kg^−1^)	26.7 ± 2.2	25.2 ± 1.6	−0.74, ±0.56 ** ↓
Propulsion Mean Force (N·kg^−1^)	20.7 ± 1.7	19.5 ± 1.2	−0.79, ±0.56 *** ↓
Unweighting Minimum Force (N·kg^−1^)	1.8 ± 1.2	1.0 ± 0.7	−0.69, ±0.57 ** ↓
Braking End Force (N·kg^−1^)	25.9 ± 2.3	25.1 ± 1.6	−0.41, ±0.59 * ↓
**Velocities**			
Braking Minimum Velocity (m·s^−1^)	1.39 ± 0.22	1.58 ± 0.13	0.95, ±0.55 *** ↑
Propulsion Peak Velocity (m·s^−1^)	2.79 ± 0.19	2.85 ± 0.18	0.32, ±0.59
**Displacements**			
Braking COM Displacement (%LL)	34.4 ± 4.6	40.9 ± 4.7	1.13, ±0.50 **** ↑
Jump Height (cm)	34.5 ± 5.7	36.0 ± 5.2	0.28, ±0.59
**Composite Variables**			
RSImod (cm·s^−1^)	50.7 ± 9.4	48.0 ± 8.0	−0.30, ±0.59
Negative to Positive Impulse (%)	37.8 ± 3.9	40.2 ± 1.7	0.74, ±0.58 ** ↑

LL = leg length; RSImod = reactive strength index-modified; COM = centre of mass. Chances that the true effect exceeds the small (ES ± 0.2) reference value are* possible, ** likely, *** very likely, and **** most likely. Direction of difference: positive ↑ and negative ↓.

**Table 2:** A comparison of discrete kinetic and kinematic CMJ variables between groups by performance.

Jump Variables	Low (*n* = 18)	High (*n* = 15)	ES (±90% CI)
	Mean ± SD	Mean ± SD	
**Phases**			
Unweighting Phase (ms)	302 ± 30	315 ± 25	0.49, ±0.57 ** ↑
Braking Phase (ms)	147 ± 19	157 ± 25	0.40, ±0.60 * ↑
Propulsion Phase (ms)	262 ± 33	262 ± 28	0.03, ±0.59
**Forces**			
Propulsion Peak Force (N·kg^−1^)	25.7 ± 2.3	26.2 ± 1.8	0.24, ±0.59
Propulsion Mean Force (N·kg^−1^)	19.5 ± 1.5	20.7 ± 1.4	0.80, ±0.54 *** ↑
Unweighting Minimum Force (N·kg^−1^)	1.4 ± 0.8	1.4 ± 1.3	−0.27, ±0.62
Braking End Force (N·kg^−1^)	25.2 ± 2.0	25.8 ± 2.0	0.30, ±0.60
**Velocities**			
Braking Minimum Velocity (m·s^−1^)	1.45 ± 0.20	1.54 ± 0.20	0.45, ±0.59 ** ↑
Propulsion Peak Velocity (m·s^−1^)	2.68 ± 0.12	2.99 ± 0.09	1.62, ±0.34 ****↑
**Displacements**			
Braking COM Displacement (%LL)	35.7 ± 5.9	40.2 ± 4.2	0.82, ±0.53 *** ↑
Jump Height (cm)	31.2 ± 3.2	40.2 ± 2.9	1.62, ±0.34 **** ↑
**Composite Variables**			
RSImod (cm·s^−1^)	44.4 ± 6.8	55.2 ± 7.0	1.22, ±0.49 **** ↑
Negative to Positive Impulse (%)	40.0 ± 2.9	37.9 ± 3.1	−0.62, ±0.58 ** ↓

LL = leg length; RSImod = reactive strength index-modified; COM = centre of mass. Chances that the true effect exceeds the small (ES ± 0.2) reference value are * possible, ** likely, *** very likely, and **** most likely. Direction of difference: positive ↑ and negative ↓.

**Table 3:** A comparison of discrete kinetic and kinematic CMJ variables between groups by performance.

Jump Variables	Bimodal Low (*n* = 8)	Bimodal High (*n* = 9)	ES (±90% CI)
	Mean ± SD	Mean ± SD	
**Phases**			
Unweighting Phase (ms)	319 ± 34	320 ± 27	0.07, ±0.89
Braking Phase (ms)	156 ± 16	157 ± 16	0.07, ±0.88
Propulsion Phase (ms)	284 ± 25	276 ± 21	−0.34, ±0.88
**Forces**			
Propulsion Peak Force (N·kg^−1^)	24.6 ± 1.6	25.7 ± 1.6	0.66, ±0.83 ** ↑
Propulsion Mean Force (N·kg^−1^)	18.7 ± 1.1	20.1 ± 0.8	1.22, ±0.73 *** ↑
Unweighting Minimum Force (N·kg^−1^)	1.1 ± 0.8	0.9 ± 0.5	−0.19, ±0.88
Braking End Force (N·kg^−1^)	24.5 ± 1.5	25.6 ± 1.6	0.67, ±0.83 ** ↑
**Velocities**			
Braking Minimum Velocity (m·s^−1^)	1.53 ± 0.15	1.63 ± 0.09	0.81, ±0.85 ** ↑
Propulsion Peak Velocity (m·s^−1^)	2.69 ± 0.15	2.99 ± 0.06	1.56, ±0.66 **** ↑
**Displacements**			
Braking COM Displacement (%LL)	39.3 ± 5.4	42.3 ± 3.6	0.67, ±0.86 ** ↑
Jump Height (cm)	31.4 ± 3.8	40.1 ± 1.7	1.59, ±0.65 **** ↑
**Composite Variables**			
RSImod (cm·s^−1^)	41.8 ± 6.6	53.5 ± 4.4	1.37, ±0.72 *** ↑
Negative to Positive Impulse (%)	41.2 ± 1.7	39.3 ± 1.2	−1.13, ±0.75 *** ↓

LL = leg length; RSImod = reactive strength index-modified; COM = centre of mass. Chances that the true effect exceeds the small (ES ± 0.2) reference value are * possible, ** likely, *** very likely, and **** most likely. Direction of difference: positive ↑ and negative ↓.

**Table 4:** A comparison of discrete kinetic and kinematic CMJ variables between groups by performance.

Jump Variables	Unimodal Low (*n* = 10)	Unimodal High (*n* = 6)	ES (±90% CI)
	Mean ± SD	Mean ± SD	
**Phases**			
Unweighting Phase (ms)	289 ± 18	308 ± 24	0.86, ±0.89 ** ↑
Braking Phase (ms)	141 ± 19	156 ± 37	0.07, ±0.88
Propulsion Phase (ms)	245 ± 30	243 ± 28	−0.34, ±0.88
**Forces**			
Propulsion Peak Force (N·kg^−1^)	26.6 ± 2.4	26.9 ± 2.0	0.15, ±0.91
Propulsion Mean Force (N·kg^−1^)	20.2 ± 1.5	21.7 ± 1.7	0.87, ±0.88 ** ↑
Unweighting Minimum Force (N·kg^−1^)	1.6 ± 0.8	2.0 ± 1.8	−0.17, ±1.18
Braking End Force (N·kg^−1^)	25.8 ± 2.3	26.2 ± 2.6	0.14, ±1.00
**Velocities**			
Braking Minimum Velocity (m·s^−1^)	1.38 ± 0.21	1.41 ± 0.25	0.11, ±1.00
Propulsion Peak Velocity (m·s^−1^)	2.67 ± 0.10	2.99 ± 0.13	1.65, ±0.62 **** ↑
**Displacements**			
Braking COM Displacement (%LL)	32.9 ± 4.7	37.1 ± 3.2	0.91, ±0.73 ** ↑
Jump Height (cm)	31.0 ± 2.8	40.4 ± 4.3	1.63, ±0.65 **** ↑
**Composite Variables**			
RSImod (cm·s^−1^)	46.5 ± 6.5	57.9 ± 9.6	1.20, ±0.82 *** ↑
Negative to Positive Impulse (%)	39.0 ± 3.4	35.9 ± 4.2	−0.78, ±0.98 ** ↓

LL = leg length; RSImod = reactive strength index-modified; COM = centre of mass. Chances that the true effect exceeds the small (ES ± 0.2) reference value are * possible, ** likely, *** very likely, and **** most likely. Direction of difference: positive ↑ and negative ↓.
